# Directed evolution of cell size in *Escherichia coli*

**DOI:** 10.1186/s12862-014-0257-1

**Published:** 2014-12-17

**Authors:** Mari Yoshida, Saburo Tsuru, Naoko Hirata, Shigeto Seno, Hideo Matsuda, Bei-Wen Ying, Tetsuya Yomo

**Affiliations:** Graduate School of Information Science and Technology, Osaka University, 1-5 Yamadaoka, Suita, Osaka 565-0871 Japan; Graduate School of Life and Environmental Sciences, University of Tsukuba, Tsukuba, Ibaraki 305-8572 Japan; Graduate School of Frontier Biosciences, Osaka University, 1-5 Yamadaoka, Suita, Osaka 565-0871 Japan; Exploratory Research for Advanced Technology (ERATO), Japan Science and Technology Agency (JST), Suita, Osaka 565-0871 Japan; Earth-Life Science Institute, Tokyo Institute of Technology, 2-12-1-IE-1 Ookayama, Meguro-ku, Tokyo 152-8550 Japan

**Keywords:** Cell size, Experimental evolution, Evolutional constraints

## Abstract

**Background:**

In bacteria, cell size affects chromosome replication, the assembly of division machinery, cell wall synthesis, membrane synthesis and ultimately growth rate. In addition, cell size can also be a target for Darwinian evolution for protection from predators. This strong coupling of cell size and growth, however, could lead to the introduction of growth defects after size evolution. An important question remains: can bacterial cell size change and/or evolve without imposing a growth burden?

**Results:**

The directed evolution of particular cell sizes, without a growth burden, was tested with a laboratory *Escherichia coli* strain. Cells of defined size ranges were collected by a cell sorter and were subsequently cultured. This selection-propagation cycle was repeated, and significant changes in cell size were detected within 400 generations. In addition, the width of the size distribution was altered. The changes in cell size were unaccompanied by a growth burden. Whole genome sequencing revealed that only a few mutations in genes related to membrane synthesis conferred the size evolution.

**Conclusions:**

In conclusion, bacterial cell size could evolve, through a few mutations, without growth reduction. The size evolution without growth reduction suggests a rapid evolutionary change to diverse cell sizes in bacterial survival strategies.

**Electronic supplementary material:**

The online version of this article (doi:10.1186/s12862-014-0257-1) contains supplementary material, which is available to authorized users.

## Background

Cell size is a key feature for all living things, from bacteria to mammals. In bacteria, cell size plays an important role in fitness, both directly and indirectly. For example, a bacterium’s vulnerability to predation by protists and host immune cells, such as neutrophils, depends on its cell size [1,2]. In addition, the cell size is relevant to mechanisms of antibiotic resistance [3] and protection from bacterial phages [4]. Thus, the bacterial cell size itself could be a target of selective pressure in the natural environment in addition to other targets, such as growth rate.

In bacteria, as well as eukaryotes, cell size is also closely related to cell proliferation. Cell size affects the uptake of nutrients from outside of the cell, the concentrations of cellular components and the progress of intracellular biochemical reactions [5]. Importantly, the initiation of chromosomal replication [6] and the assembly of the division machinery [7] also depend on cell size through underlying molecular mechanisms, resulting in homeostatic and recursive reproduction of an optimal cellular state [8]. These facts show that the bacterial cell size is strongly coupled with its growth rate.

Such a strong coupling between the cell size and the growth rate could play two contradicting roles in evolution. Such coupling may facilitate adaptation during evolution. For example, a rapid growth state could be achieved as a consequence of the selection in size and vice versa. However, coupling that is too strong may introduce conflicts during evolution in cell size. For instance, if large cell size is favorable in the environment, for example to avoid grazing pressure from predators, but not for the fast-growing states, then the cells will face fitness conflicts, called evolutionary trade-offs [9]. Similar concerns regarding the coupling between other traits, such as gene expression responsiveness across conditions (plasticity) and cell-to-cell variation (noise), have been discussed previously [10].

Consistent with the possible conflicts between cell size and growth rate, most cell size mutants exhibit defective growth rates [11-15]. Previous studies have identified several mutations that introduce abnormal morphology and volume. For example, when the genes related to cell division, cell wall synthesis and membrane synthesis are inactivated, the mutant cells exhibit different shapes and sizes from the wild-type [14,15]. In contrast, mutants with defective lipid biosynthesis cannot synthesize the cell body rapidly and become smaller [13]. Although these kinds of mutations might contribute to size evolution, such induced mutants face the conflict of growth rate instead.

Is bacterial cell size evolvable in several directions, from small to large, without an added growth burden? If so, how many and what kind of mutations are required? Unlike the analysis of artificially constructed mutations for a limited number of target genes, experimental evolution of microbial populations could be useful for identifying such genetic paths across the whole genome. The previous experimental evolution studies suggest that cell size could evolve along with growth adaptation. Lenski’s group performed the serial transfer of *Escherichia coli* over thousands of generations [16]. As an evolutionary consequence, the cells obtained not only faster growth speed than the ancestors but also larger size [16,17], even in the absence of explicit directional selection on the cell size.

To explore the directed evolution toward different cell sizes, directed evolution experiments to finite cell sizes were required. Because the previous long-term experimental evolution with serial passages lacked explicit size selections, the selection target and its pressure were uncontrolled, allowing for the accumulation of mutations unrelated to size changes. It is unclear how rapidly cell size can evolve in the presence of explicit size selections. Thus, directed evolution experiments with a tunable selection for cell size within fewer generations are desirable.

Here, using *E. coli*, we performed evolution experiments on cell size for a short period to address whether cell size evolved in response to the size selections without growth conflict. We employed a cell sorter to directly select specific cell sizes that were smaller than the ancestor. Two target sizes were repeatedly selected, along with the size distributions, mild and severe. The former target size selected cells that were slightly smaller than the ancestors (1% of the cells around the peak of the size distributions). The latter target size selected cells with far smaller sizes (the smallest 1% of the cells). The sorted cells were cultured overnight until the next size selections. Within 400 generations, smaller mutants were selected in response to each size selection. The growth rates of these mutants did not decrease. Whole genome sequencing revealed a few genomic mutations, as expected. We found that only a few mutations in the genes related to membrane synthesis could confer size evolution without growth conflict. We also tested the directed evolution toward larger cell sizes. The bacterial size evolution without growth reduction suggests that the rapid evolutionary change to diverse cell sizes represents a survival strategy.

## Results and discussion

### Bacterial cell size distributed broadly in a clonal population

**Figure 1:**
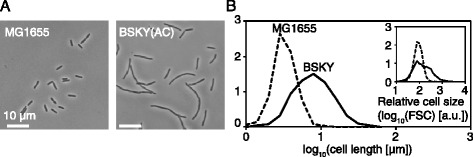
**Cell size distributions of two** ***E. coli*** **strains. (A)** Phase-contrast images of two *E. coli* strains (left, MG1655, and right, BSKY). The scale bar represents 10 μm. **(B)** Cell size distributions obtained by microscopy. The solid and dashed lines indicate BSKY and MDS42, respectively. The inset represents the corresponding cell size distributions obtained by flow cytometry.

We employed a GFP-integrated derivative of *E. coli* DH1, called BSKY, as an ancestral clonal population. DH1, including BSKY, is large, filamentous and rod-shaped and is more heterogeneous in size than the wild-type strain, MG1655 (Figure 1). This property implies that BSKY has a capacity to evolve to smaller sizes by reducing the filamentous fraction in response to the appropriate selections without facing physical limitations. Therefore, we considered this strain an appropriate ancestor to test whether the evolution to smaller size is accompanied with growth changes. We used a fluorescence activated cell sorter (FACS) to sort the bacterial cells according to their relative size, based on the forward scatter value (FSC) in flow cytometry (Figure 1B inset). The FSC basically reflects the length, or the longest diameter, in rod-shaped bacteria, and agrees well with microscopic observation [18]. As a result, the larger and/or broader size distributions were also captured consistently in flow cytometry and microscopy. We employed mean values and standard deviations on a logarithmic scale to characterize these size distributions.

### Repeated cycles of size selection was examined with population propagation

**Figure 2:**
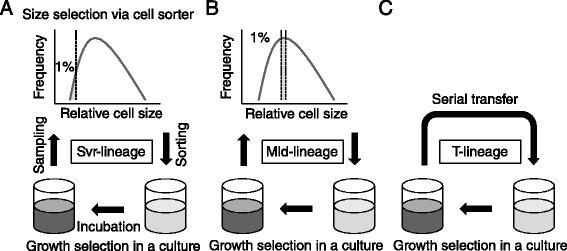
**Schematics of the experimental evolution strategies.** Rounds of directed size evolution consist of size selections using a cell sorter and subsequent growth selections in cell culture. The size selections were defined as the smallest 1% of the population (Svr-lineage, **(A)**) and that around peak (Mld-lineage, **(B)**). As a control, the T-lineage consists of growth selection without size selection **(C)**. The detailed procedure is described in the main text.

**Figure 3:**
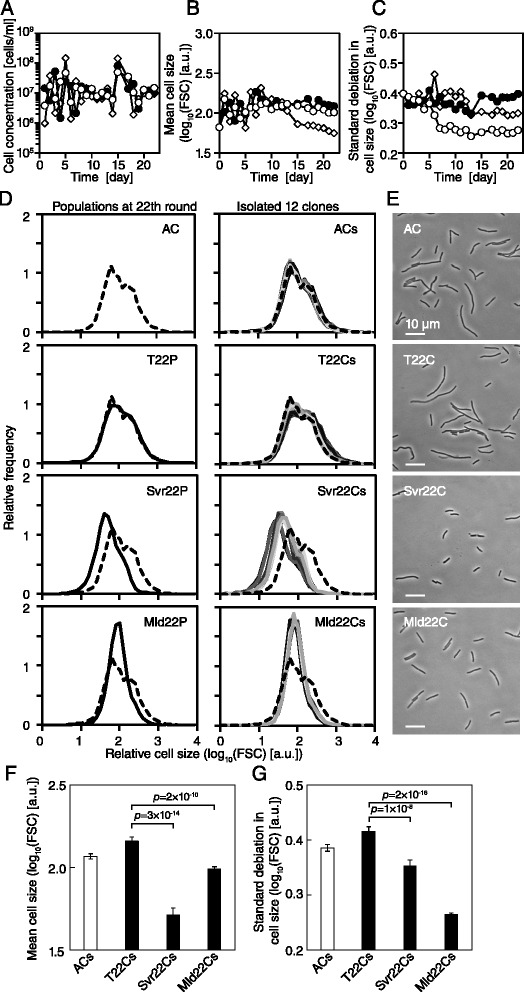
**Evolution process and final populations of evolved lineages.** The trajectory of the cell concentration **(A)**, the mean cell size **(B)** and the standard deviation **(C)** under the size selection process. Open diamond, open circle and closed circle represent the Svr-, Mld- and T-lineages, respectively. **(D)** Cell size distributions of the population of the final round (left) and the isolated 12 clones (right), where the isolates are distinguished by gray-scaled lines. The top two panels represent ancestral clones. The other panels correspond to the T-, Svr- and Mld- lineages at the bottom. The insets represent the Cell IDs in Table 1. All data were obtained at approximately 10^7^ cells/ml. The dotted line indicates one of the AC isolated clones (top left). **(E)** Phase contrast images of the isolated clones are shown. The insets also represent the Cell IDs in Table 1. The white bars indicate 10 μm. **(F)** The mean cell size of the 12 isolates. **(G)** The standard deviation in cell size of the 12 isolates. The error bars are 95% confidence limits. *P*-values are for t-test (N = 12).

Starting from a genetically identical cell population of BSKY, we tested the possibility of evolution toward a smaller cell size through size selections, where the strength of the selection was examined in 2 ways (Figure 2A and B). Our experimental rounds consist of two simple selections, size selection via FACS and growth selection in a culture. The cells whose size met the selection criteria represented greater fitness in the size selection, and faster-growing cells naturally outcompeted slow-growing cells in the cultures. The cells were sampled from the overnight culture, and the particular fractions exhibiting the target sizes were sorted to fresh medium using FACS. The size selections were examined in the smallest 1% of the cells (severe selection) and around the peak (mild selection) to yield the Svr- and Mld-lineages, respectively. The numbers of the sorted cells were decided based on the growth rate of the previous round, so they reached approximately 10^7^ cells/ml after overnight culturing. The typical values were 20 to 2000 cells in 1 ml of fresh medium. Consistent with the small population sizes, the cell concentrations fluctuated day by day, even in the general serial transfer cells (T-lineage) (Figure 3A). These rounds were repeated daily, in parallel with the T-lineage, which was not sorted by size using FACS and used as a control (Figure 2C). More detailed procedures are described in the Methods section.

### Bacterial cell size decreased, and homogeneity increased

During the selection cycles, we observed changes in the size distributions (Figure 3B and C). In the Svr-lineage, the mean cell size decreased after 15 days (190 generations), achieving a 2.2-fold reduction compared with the T-lineage at the final day (288 generations) (Figure 3B). In contrast, the mean cell size in the Mld-lineage showed only a slight reduction. These results are consistent with the strength of the size selection. We also found that variation in the Mld-lineage started to decrease after 4 days (57 generations), relative to the Svr- and T-lineages (Figure 3C).

After 22 days (or 22 rounds), we obtained cell populations from each lineage (T22P, Svr22P and Mld22P). In addition, we isolated 12 clonal populations from each lineage (T22Cs, Svr22Cs and Mld22Cs, Table 1). We then compared the distributions of these populations with that of the ancestral population (AP) and its clones (ACs) (Figure 3D). The distributions of these isolates were obtained directly from the final overnight cultures, without another FACS-mediated size selection (see the Methods section). T22P exhibited a similar size distribution to ACs and isolates. T22Cs also kept their size distributions. This finding indicates that the size distribution, in the absence of size selections, remained constant in the presence of the growth selections through hundreds of generations. In contrast, we found that all clones in the Svr- and Mld-lineages (Svr22Cs and Mld22Cs) evolved in size, as designed by the size selections (Figure 3D–G), as did the distributions of Svr22P and Mld22P. That is, the distributions of Svr22Cs revealed greatly reduced mean cell sizes and those of Mld22Cs were slightly reduced (Figure 3F). Both lineages evolutionarily reduced the filamentous subpopulation. These results also infer that cell size may show a maternal-like heritability, since small cells should tend to produce small progeny, which is similar to the cells with the old pole grow more slowly [19].

**Table 1:** **Cell IDs of the experiments**

**Cell IDs**	**Descriptions**
AC	Ancestral clonal population of BSKY
ACs	12 clones isolated from AC
AC-T5	Population propagated for 5 days without size selection from an AC
T22P	Population obtained at the 22nd round in the T-lineage
T22Cs	12 clones isolated from T22P
T22P-T5	Population propagated for 5 days without size selection from an T22C
Svr22P	Population obtained at the 22nd round in the Svr-lineage
Svr22Cs	12 clones isolated from Svr22P
Svr22P-T5	Population propagated for 5 days without size selection from an Svr22C
Mld22P	Population obtained at the 22nd round in the Mld-lineage
Mld22Cs	12 clones isolated from Mld22P
Mld22P-T5	Population propagated for 5 days without size selection from an Mld22C
L22P	Population obtained at the 22nd round in the L-lineage
L22Cs	12 clones isolated from L22P
L22P-T5	Population propagated for 5 days without size selection from an L22C
Ls8P	Population obtained at the 8th round in the Ls-lineage
Ls8Cs	12 clones isolated from Ls8P
Ls8P-T5	Population propagated for 5 days without size selection from an Ls8C

We also found that the variation in both the Svr- and the Mld-lineages decreased but to different extents (Figure 3G). The variation in Mld22Cs greatly decreased, while that in Svr22Cs decreased slightly. The selection around the peak may have stabilized the size distribution, reducing the variation in size. These evolved properties were confirmed by direct microscopic observation (Figure 3E, Additional file 1: Figure S1 and Additional file 2: Figure S2). These results indicate that the bacterial cell size could evolve through the simple selection process, not only in average but interestingly also in cell-to-cell variation, even in the clonal population.

### The evolved cells maintained small size, independent of cell concentrations

**Figure 4:**
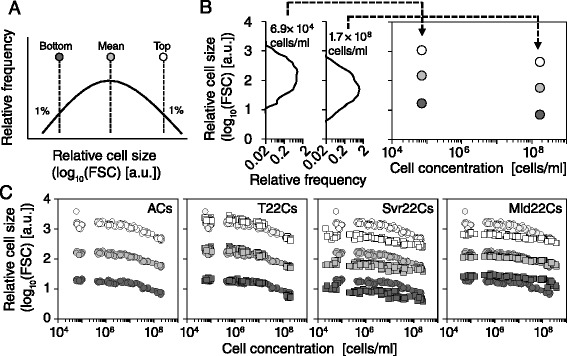
**The cell concentration dependency of cell size. (A)** The reference lines of the cell size distribution. The dark gray, light gray and open symbols indicate the bottom 1%, mean and top 1% of the size distributions, respectively. **(B)** These representative images plot three references (bottom, mean and top) over cell concentrations. The left two panels represent the size distributions for an ancestral clone obtained at different cell concentrations in the same growth culture; the concentrations are indicated in the inset. The three references are plotted in the right panel along with the corresponding cell concentrations, as indicated by the arrows. **(C)** Shown are the references of the cell size distributions over different cell concentrations for the isolated 12 clones from the ancestor (far left), T-lineage (2nd from the left), Svr-lineage (2nd from the right) and Mld-lineage (far right) populations. Each panel includes the distribution of ACs (circle), while the other clones are indicated by squares.

Because the size selections were designed to work on the cell size at particular cell concentrations and particular fractions of the distributions, they cannot reveal whether the evolved traits occurred at different cell concentrations or outside of the mean (Figures 2 and 4). In fact, cell size can change with time, cell concentration and/or growth phases. Bacterial cell size begins to decrease after the late log phase of growth [20]. Thus, we explored the size distribution of 12 clones at different cell concentrations along with the growth curves. For each distribution, at each time point, we analyzed by multipoint monitoring the mean, the top 1% and the bottom 1% lines (Figure 4A), where these reference lines were thresholded in size selections. These three references were plotted over various cell concentrations (Figure 4B and D). For all clones, as expected, including the top and bottom lines, the mean cell size of the ancestors started to decrease as the cell concentrations increased, long before the cultures left the exponential growth phase (far left in Figure 4C). T22Cs showed a similar dependence on the cell concentration to the ancestral clones at each point (2nd from the left in Figure 4C).

In contrast, all evolved clones in the Svr- and Mld-lineages showed their evolved properties below 10^8^ cells/ml and had different dependencies on cell concentration. The cell size of Svr22Cs was small in mean value, as were both the top 1% and the bottom 1% lines (2nd from the right in Figure 4C) and became slightly insensitive to cellular concentrations, relative to the ancestor clones. Mld22Cs notably became dense around the mean and maintained their cell size over various cell concentrations (far right in Figure 4C). Thus, size selection at a particular cell concentration promotes size evolution at the different cell concentrations.

We further tested whether size selections are necessary for maintaining these evolved traits. To answer this question, we randomly chose 12 isolates from each lineage and propagated them for 5 days in the absence of size selections. All isolates maintained the corresponding traits in size over different cell concentrations (Additional file 3: Figure S3).

### Whole genome resequencing revealed a few mutations in growth-related genes

To understand what kinds of mutations cause size evolution, we examined whole genome resequencing for all 4 populations (AC, T22P, Svr22P and Mld22P, Table 2). We identified only one SNP, which was found in the coding region of phosphatide cytidylyltransferase, *cdsA*, in Svr22P. This SNP is a nonsynonymous mutation resulting in an amino acid substitution from Glu to Lys at the 109th residue. In Mld22P, we also found a single base insertion in the coding region of transketolase, *tktA*. This insertion is also nonsynonymous but with a frameshift from Lys658. Considering the mutation rate (5.4 × 10^−10^ per bp per generation [21,22]), the genome size (4.6 × 10^6^ bp) and the number of generations (200–400), the evolved population was expected to have 5.4 × 10^−10^ × 4.6 × 10^6^ × (200–400) ~ 0.5–1 mutations per individual. This value agreed rationally with the small number of mutations contributing on the cell size changes. Therefore, we assumed these few mutations per individual are appropriate candidates to inhibit filamentation. The effective population size given by the harmonic mean of the transfer sizes (200–2000) and final population sizes (1 × 10^7^–1 × 10^8^) each day was approximately 200–2000, where the final population sizes each day were shown in Figure 3A. Therefore, 0.5–1 mutations per individual multiplied by 200–2000 (individuals) give 100–2000 mutations in a population without selection. These estimations support that the mutations which alter cell size were relatively frequent, suggesting the rapidness of the cell size evolution in typical bacterial populations with large population sizes.

**Table 2:** **Mutation list**

**Lineage**	**Gene**	**Position (bp)**	**BPS/InDel**	**Amino acid substitutions**
Svr-lineage	*cdsA*	3675204	BPS (G to A)	Glu109Lys
Mld-lineage	*tktA*	804101	Insertion (AG to AAG)	Frameshift from Lys658

BPS: base pair substitutions, InDel: insertions and deletions.

**Figure 5:**
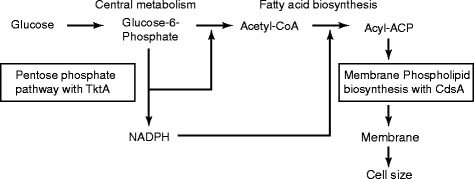
**Roles of targeted genes in membrane synthesis.** Two genomic mutations were identified in the genes *tktA* and *cdsA*, which are involved in membrane synthesis from glucose metabolism. In the metabolic flow from glucose to membrane synthesis, TktA is involved in the pentose phosphate pathway to produce NADPH, which is required for fatty acid synthesis. Using fatty acids, CdsA is involved in membrane phospholipid synthesis, followed by membrane construction, which affects cell size.

These two genes engage in different pathways, *cdsA* for phospholipid biosynthesis and *tktA* for the pentose phosphate pathway (Figure 5). *cdsA* is essential for cell growth because the coded enzyme provides phospholipids to build up the cell membrane. Therefore, if the SNP makes *cdsA* defective, the small cell size of the mutants is functionally relevant. A recent study reported that a mutant defective in fatty acid biosynthesis, which is the upstream pathway of phospholipid biosynthesis, exhibited a small cell size relative to MG1655 [13]. The role of *tktA* is not essential but links many important pathways for growth, such as fatty acid biosynthesis, aromatic amino acid biosynthesis, aromatic vitamin biosynthesis, nucleic acid biosynthesis and glycolysis. Upregulated *tktA* promotes the production of acetyl-CoA and NADPH, with an increase in cell mass [23]. In addition, the expression level of *tktA* is downregulated in the stationary growth phase, possibly by a sigma factor, *rpoS*, which is associated with small cell size [24]. Assuming that the downregulation or deficiency of *tktA* inhibits fatty acid biosynthesis through a lack of NADPH, a mutant defective in *tktA* could then fail to build cell membrane in a similar way to defective *cdsA*.

### Cell size evolution is not associated with a growth disadvantage

**Figure 6:**
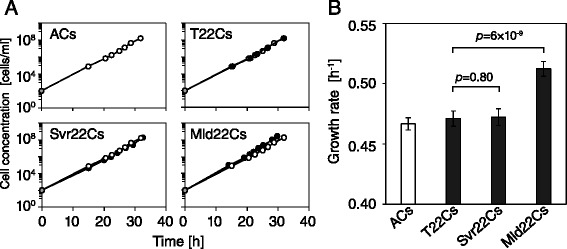
**Growth characteristics of evolved clones. (A)** The averaged growth curve of the 12 clones isolated from the ancestor (left top), the T-lineage (right top), the Svr-lineage (left bottom) and the Mld-lineage (right bottom) populations. The gray circles at time zero indicate the estimated cell concentration from the inoculation procedure. Each panel includes the growth curve of ACs (open circle), while the other clones are indicated by filled black circles. The error bars represent the standard deviations, and most bars are very small. **(B)** The growth rate of the 12 isolated clones for the exponential growth phase. The growth rate is calculated from the slope of the growth curves during the exponential growth phase. The error bars are 95% confidence limits. *P*-values are for t-test (N = 12).

If the cell size of the ancestral population was preliminarily evolved or optimized for growth, size selection may perturb it as the result of evolutionary trade-offs. As noted above, the observed growth rate of the population in the selection rounds might be biased by the initial size selections. Therefore, we explored the growth characteristics of all of the evolved clones without size selections (Figure 6). Interestingly, the growth rate of Svr22Cs was almost the same as ACs or T22Cs, within the log phase. We also found that Mld22Cs grew faster than the ancestors regardless of the presence of size selections (Figure 6B). These results indicate that the evolution in mean cell size was not necessarily associated with a growth burden. That is, the cell size evolution could be achieved without strong constraints, such as trade-offs between cell size and growth rate. This conclusion supports a loose coupling of the cell size and the growth rate, as previously observed in long-term size evolution in Lenski’s group [16].

### The rapid evolution of cell sizes implies survival strategies in the natural environments

Bacterial cell size plays important roles in survival under varying environmental conditions, such as nutrient availability and predation [5]. For example, some bacteria of different phyla employ the strategy of size reduction or filamentation to protect from predation by protists [2,25]. The observed short-term evolutionary change to diverse sizes may contribute to sustainability through a fluctuating grazing pressure or nutrient availability in the natural environment. Because trophodynamics, based on the grazing and nutrient availability, are quite complex in the wild, size evolution might be important or even advantageous. Previous field surveys explored size polymorphism within some genotypes [26,27]. Microbes may accomplish total fitness via tuning their size distribution to solve complex trophodynamics.

### Directed evolution toward larger size can occur without growth defects

Is it possible to evolve toward larger size without growth defects, just like the evolution to smaller size? Some bacterial strains exhibit large filamentous morphotypes that contribute to antipredation strategies. By applying the sorting gate for large-sized cells, the directed evolution toward larger size can be tested. In principle, filamentous morphotypes could be achieved in two extreme ways: a chained form of several cells without increasing each cell’s volume and an elongated form of single cells with a resulting increase in cell volume. Both types were observed in several bacteria, in certain conditions. Due to the technical limitations of the cell sorter, these two types were indistinguishable through light scattering patterns. Therefore, the outcome of the evolution might depend on growth competition between the two types. Nevertheless, we tried to introduce evolution to large-sized, filamentous cells without growth defects (L-lineage, Additional file 4: Figure S4 and Additional file 5: Figure S5A–C). After 22 days, the size distributions were unchanged from that of the ancestor (Additional file 5: Figure S5D). Microscopic observation, however, revealed that the chained form accounted for most of the longer cells (Additional file 5: Figure S5E). FM4-64 membrane staining showed several membrane septa between each cellular compartment in a single filament (Additional file 6: Figure S6). Moreover, the individual cell sizes within a filament remained unchanged. Thus, the simplest selection regime introduced the filamentous morphotype, but failed to introduce an evolution to larger volume between the septa, within a short time frame.

In addition to the instrumental limitation in selecting large cell sizes in a filament, one possible reason for the outcome is that a chained form with many dividing septa provides a growth advantage immediately after cell sorting versus the singular, elongated form. To avoid such putative growth competitions induced by the sorting inaccuracy and to eliminate the delusive morphotypes, we examined an alternative selection regime (Ls-cycle, Additional file 7: Figure S7). In addition to the growth selection during propagation, the revised method consisted of single-cell sorting for the populations with the largest cell size. The single-cell sorting could isolate a possible mutant from the chained forms, which are prone to divide immediately. A possible limitation of this method, however, is that the single-cell bottleneck might allow incidental fixation of growth-defective mutants relative to ancestors by genetic drift. After 8 days, however, the cells exhibited larger size distributions without growth defects during the exponential phase, before accumulating deleterious mutations (Additional file 8: Figure S8 and Additional file 9: Figure S9). Microscopic observations revealed a long, filamentous shape, composed of elongated cells in most cases (Additional files 1, 2 and Additional file 8: Figure S8F). Some filaments contained membrane septa, but the intervals between the septa were wider than that of the cells in the L-lineage, which is consistent with the elongation of each cell. Thus, the revised selection regime in the Ls-lineage drove the evolution to larger size within 139 generations without growth defects.

The clonal isolates from the evolved population in the Ls-lineage also exhibited large size (Additional file 8: Figure S8E). In addition, these isolates kept their large size after 5 days (80–90 generations) of propagation in the absence of size selections (Additional file 3 and Additional file 10: Figure S10). These results indicate that the evolved large size was inheritable. Unfortunately, whole genome resequencing detected no significant base pair substitutions nor small insertions and deletions. Thus, further analysis or superior techniques would be needed to detect possible genetic signatures.

## Conclusions

Starting from a clonal *E. coli* population, the directed evolution of cell sizes without inducing a growth burden was tested empirically. Cell size evolved to small within 400 generations in response to directed size selections. Severe selection led rapidly to a small cell size, more so than mild selection. In addition to the mean cell size, the width of the size distribution also evolutionally changed. Importantly, the cell size evolution were unaccompanied by disadvantages to the cells’ growth rate in the absence of the size selections. In conclusion, these data indicate that bacterial cell size could evolve, by means of a few mutations, in response to size selection, without strong constraints due to trade-offs of growth rate, suggesting that the rapid evolutionary change to diverse cell sizes is important for bacterial survival strategies.

## Methods

### Bacterial strains

We constructed the *E. coli* derivative strain from DH1, DH1Δ*galK::* Plac-*gfpuv5*-Pkan-*kan,* called BSKY. To construct BSKY, we used the plasmid pLacGK (pBR322 derivative) containing the *lac* promoter (Plac), its operator, the derivative gene of gfp (*gfpuv5*), kanamycin resistance gene (*kan*) and its promoter (Pkan). The corresponding fragment, Plac-*gfpuv5*-Pkan-*kan*, was flanked by an rrnBT1T2 terminator (upstream of Plac) and a t7 terminator (downstream of kan). The construction of pLacGK was reported previously [28]. The fragment of Plac-*gfpuv5*-Pkan-*kan,* including the terminators of both ends, was amplified from pLacGK with the primers T2-f (5′-aagcagaaggccatcctgacgga-3′) and T7-R2 (5′-atccggatatagttcctcctttga-3′) and was inserted into the *galK* gene on the plasmid pT0 for subsequent genomic recombination, as described previously [29]. Using the primers chgalKl (5′-aagcccacgttttacggatc-3′) and chgalKr (5′-ggcccgccgtgcagctggtt-3′), this plasmid was employed as a template for PCR of the target sequence Plac-*gfpuv5*-Pkan-*kan,* flanked by the homologous sequences of *galK*. The amplified fragment was used for genome replacement at the chromosomal location of *galK* in DH1 [29]. The final recombinant, DH1Δ*galK::*Plac-*gfpuv5*-Pkan-*kan*, was called BSKY.

### Culture conditions

Bacterial cells were grown in a minimal medium, modified M63 (mM63) that contained 62 mM K_2_HPO_4_, 39 mM KH_2_PO_4_, 15 mM (NH_4_)_2_SO_4_, 2 μM FeSO_4_ · 7H_2_O, 15 μM thiamine hydrochloride, 203 μM MgSO_4_ · 7 H_2_O and 22 mM glucose [29] and was supplemented with 100 μM kanamycin (Km) and 100 μM isopropyl β-d-1-thiogalactopyranoside (IPTG). Ancestral cells were cultured at 37°C for several passages until the growth rate stabilized and were cloned before use for experimental evolution. Other culture conditions are detailed elsewhere, where relevant to the other experimental parameters.

### Flow cytometry

Relative cell size, GFP fluorescence and cell concentration was evaluated using a flow cytometer (FACSAria cell sorter; Becton Dickinson) equipped with a 488-nm argon laser. Relative cell size was measured by the detector for the forward-scattering light, while GFP fluorescence was collected through a 515–545 nm emission filter (GFP). The GFP fluorescence was used to distinguish the cells from debris. The flow data were analyzed by scripts written in R [30]. Systematic errors, resulting from events that occurred at the bottom or top of the instrument’s range, were eliminated. Cell samples, mixed with known concentrations of fluorescent beads (3 μm Fluoresbrite YG Microspheres; Polysciences), were loaded to calculate the cell concentrations. For cell sorting, the cells of particular sizes were sorted according to their forward-scattered light intensity. To correct the FSC measurements from daily variation in instrumental condition, we calibrated the measured data by daily measurements of four different beads with known diameters: 0.75 μm for Fluoresbrite Plain YG 0.75 micron Microspheres (Polysciences), 1.0 μm for Fluoresbrite calibration grade 1.0 micron YG Microspheres (Polysciences), 2.0 μm for Latex Microsphere Suspensions (Duke Scientific Corporation) and 3.0 μm for Fluoresbrite calibration grade 3.0 micron Microspheres (Polysciences).

### Evolutionary experiment

Starting from a genetically identical cell population of BSKY, we conducted experimental rounds consisting of two simple selections: size selection via FACS and growth selection in culture. We prepared the cells for the evolutionary experiments by overnight culture. Through the evolutionary experiment, we sampled the overnight culture, and the particular fractions of the population exhibiting the target sizes were sorted to the fresh medium using FACS. The size selections were examined in the smallest 1% of the population (severe selection) and around the peak (mild selection), (Svr- and Mld-lineage, respectively). The numbers of the sorted cells were calculated from the growth rate of the previous round, so they would reach approximately 10^7^ cells/ml in the next overnight culture. The typical values were 20 to 2000 cells in 1 ml of fresh medium. These cycles were repeated daily, along with the general serial transfer line without the size selection. We stored each sampled population at −80°C for later experiments. Every day, we calculated the number of generations per day (*g*) as the following equation: *g =* log_2_(N_t+Δt_/N_t_), where N_t_ is the initial cell concentration and N_t+Δt_ is the final cell concentration in the cell culture. We used the number of sorted cells as N_t_, while N_t+Δt_ was measured by flow cytometry as described above. The total number of generations was calculated by summing *g*.

### Single clone assay experiment

Cells from freezer stocks were plated on mM63 agar and incubated at 37°C for 4 days. Twelve colonies were picked from each strain and suspended in mM63 medium. Then, they were stored at −80°C with glycerol. Clonal isolated cells were inoculated into mM63 medium from freezer stocks. The initial cell concentration was 10^3^ cells/ml, and the cells were incubated for 20 hours. These cultures were then diluted, and 10^2^ cells were transferred to 1 ml of fresh medium. The cells were sampled every 2–3 hours. The growth rates (Malthusian parameter) during the exponential growth phase were calculated from the slopes of the growth curves according to the standard Malthusian growth model.

### Genomic DNA preparation

Glycerol-stock cells were inoculated into mM63 medium and grown until OD_600_ = 0.5 at 37°C. The cell cultures were subsequently diluted to OD_600_ = 0.05 with fresh medium, and grown to stationary phase. Rifampicin (final concentration 300 μg/ml) was subsequently added, and the culture was continued for another 3 hours to block the initiation of DNA replication. The cells were collected by centrifugation at 25°C at 5000 × *g* for 5 min, and the pelleted cells were stored at −80°C prior to use. Genomic DNA was extracted using standard procedures, following instructions from the Aqua Pure Genomic DNA Isolation kit (Bio-Rad) and Wizard Genomic DNA Purification kit (Promega). Then, we stored the genomic DNA at −20°C.

### Whole-genome resequencing

The genomic DNA library was prepared for Roche 454 sequencing using emulsion PCR kits (GS Junior Titanium emPCR Kit (Lib-L), Roche). Whole-genome sequencing was performed on the Roche 454 GS Junior with the genomic DNA library (average read length of 386 bp; average per-site coverage of 5.2; average coverage of 98% of the genome per strain) with the appropriate kits (GS Junior Titanium Sequencing Kit and GS Junior Titanium PicoTiterPlate Kit, Roche). These preparation and analyses were examined using the Genome Information Research Center (GIRC) at Osaka University (Japan). Using the GS Reference Mapper software (ver. 2.6; Roche), these reads were then aligned onto the *E. coli* DH1 reference chromosome (Accession: CP001637, Version: CP001637.1, GI: 260447279, Size: 4,630,707 bp) to identify putative mutations. Candidate mutations were detected as ’HC (High Confidence) Differences,” “HC Structural Rearrangements” and “HC Structural Variants” by the software with recommended parameter settings (system default: Seed step: 12; Seed length: 16; Seed count: 1; Hit-per-seed limit: 70; Minimum overlap length: 40; Minimum overlap identity: 90; Alignment identity score: 2, Alignment difference score: −3; Repeat score threshold: 12). As detailed in the commercial manual, confidence was determined by the built-in algorithm in the software, where high-confidence was determined along with the following three rules: There must be at least 3 non-duplicate reads with the difference (1); There must be both forward and reverse reads showing the difference (2); If the difference is a single-base overcall or undercall, then the reads with the difference must form the consensus of the sequenced reads and the signal distribution of the differing reads must vary from the matching reads (3). All high-confidence SNP sites and candidates for DIP variations (deletions, insertions and inversions) were checked by capillary Sanger sequencing of PCR products amplified directly from the genome.

### Microscopic observations

Cells in the exponential growth phase (10^6^ cells/ml, 10 μl of the culture) were placed on a thin agarose pad (1.5%), and the pad was subsequently placed down on a glass dish, resulting in a monolayer of cells between the agarose pad and glass dish. The culture condition was the same as the single colony assay experiment. Images were acquired at 60× magnification using a fluorescence microscope (TE2000; Nikon) and a cooled CCD camera (DV887; Andor). The gain of the camera was 100, and exposure time was 50 ms. The images were analyzed using ImageJ software (NIH) to measure cell length.

## Availability of supporting data

The data sets supporting the results of this article are included within the article and its additional files.

## Additional files

Additional file 1: Figure S1. Microscopic pictures of size-evolved cell populations. Each column corresponds to each cell population, as indicated in the top panels (AC, T22C, Svr22C, Mld22C L22C and Ls8C from left to right). These Cell IDs are explained in Table 1. Scale bars represent 20 μm. Additional file 2: Figure S2. Cell length distributions obtained from microscopic observation. Cell IDs are indicated in the insets. Dashed lines represent the distribution of AC (identical to top left panel) for reference. Additional file 3: Figure S3. The cell concentration dependency of cell size after daily serial transfers for 5 days without size selection. Twelve isolates from each evolved lineage (ACs, T22Cs, Svr22Cs, Mld22Cs, L22Cs and Ls8Cs) were propagated every day in the absence of size selection. In each propagation, 100 cells were transferred to 1 ml of fresh medium for 5 days (over 80 generations). The obtained populations are denoted as ACs-T5, T22Cs-T5, Svr22Cs-T5, Mld22Cs-T5, L22Cs-T5 and Ls8Cs-T5, as shown in the insets of the panels. (A) The mean growth curves of the transferred populations. Error bars indicate the standard deviation among the 12 isolates. The gray circles at time zero indicate the initial cell concentrations calculated from inoculation procedure. Each panel includes the growth curves of ACs (open circle), while the other clones are indicated by filled black circles. (B) The size references of the cell size distributions over different cell concentrations. Each panel includes the references of ACs (circle), while the other clones are indicated by squares. The dark gray, light gray and open symbols indicate the bottom 1%, mean and top 1% of the size distributions, respectively. Additional file 4: Figure S4. Schematics of the experimental evolution toward a large cell size. Rounds for the directed size evolution consist of the particular size selections via cell sorter and the subsequent growth selections in a culture. The size selections were examined according to the fraction containing the largest 1% of cells (L-lineage). As a control, T-lineage consists of growth selection without size selection (as described in the main text). We determined the numbers of the sorted cells from the growth rate of the previous round, so they would reach approximately 10^7^ cells/ml overnight. Additional file 5: Figure S5. Evolutionary process toward a large cell size. The trajectory of the cell concentration (A), the mean cell size (B) and the standard deviation (C) under the size selection process is shown. Open circles and closed circles represent the L- and T-lineages, respectively. (D) Cell size distributions of the population of the final round (left) and the isolated 12 clones (right), where the isolates are distinguished by gray-scaled lines. The top two panels represent ancestral clones. The other panels correspond to the T- and L-lineages at the bottom. The insets represent the Cell IDs in Table 1. All data were obtained at approximately 10^7^ cells/ml. The dotted line indicates one of the AC isolated clones (top left). (E) Phase contrast images of the isolated clones. The insets also represent the Cell IDs in Table 1. The white bars indicate 10 μm. The other images are shown in Additional file 1: Figure S1. Additional file 6: Figure S6. Dividing septa of size-evolved cells. Phase contrast and fluorescence images of ACs (A), L22Cs (B) and Ls8Cs (C) are shown, where the Cell IDs are noted in Table 1. Cell membranes and DNA were stained with FM4-64 and Thiazole Orange (TO), respectively. For cell staining, cell cultures supplemented with 10 mM EDTA, 0.1% (v/v) Tween-20 and 420 pM of TO (BD Biosciences) were incubated for 15 min at room temperature. The cultures were further incubated for 1 min at room temperature after addition of 5 μg/ml of FM4-64 (Invitrogen). In each cell, bright field images (BF), red fluorescence images for FM4-64, green fluorescence images for TO and merged images (merged) were obtained (from left to right). The scale bars represent 20 μm. Additional file 7: Figure S7. Schematics of the experimental evolution to large cell size through a single-cell bottleneck. Seventy-two single cells in the largest 1% fraction in the population were sorted individually into the fresh medium (1 cell/ml) using 24-well plates. The cell cultures were incubated until the cell concentrations surpassed 10^4^ cells/ml, which is the instrumental detection limit for cell number. The incubation time was 1.5 days until the 6th round and 2 days beyond that. The cell cultures for the subsequent cell sorting were selected among grown cultures based on which culture has the largest 1% size. Typically, approximately one-third of the cultures reached a sufficient cell concentration to be analyzed for the size selections in every round. The cells resulting from cycles toward the larger size through a single-cell bottleneck was called Ls-lineage. Additional file 8: Figure S8. Time-series behaviors in the selection cycles toward larger size through a single-cell bottleneck. The mean and standard deviation of the size distributions during the evolution process for the Ls-lineage (triangles) were measured (A and B), and the growth rates and the cell concentrations of the cultures were also calculated (C and D). The other circles are denoted in Additional file 7: Figure S7. The cell population at the final round (8th round) and its 12 isolates were analyzed to obtain the size distributions (E). The dotted line indicates one of the AC isolated clones in Additional file 5: Figure S5. The microscopic images of the evolved clone are shown with that of the ancestor (F). The scale bars represent 20 μm. The insets represent the Cell IDs in Table 1. Additional file 9: Figure S9. Growth characteristics of the evolved populations toward larger size. (A) The averaged growth curves of the isolated 12 clones from L-lineage (L22Cs) and Ls-lineage (Ls8Cs). The Cell IDs are indicated in the inset. Open circles represent the ancestral clone (ACs). The gray circles at time zero indicate the estimated cell concentration from inoculation procedure. The error bars represent the standard deviation. (B) The growth rate of the isolated 12 clones for the exponential growth phase. The bars for ACs and T22Cs are shown for reference. The error bars represent 95% confidence intervals. *P*-values are for t-test. Additional file 10: Figure S10 The cell concentration dependency of cell size in the evolved populations toward larger size. The size references defined in Figure 4A of the cell size distributions over different cell concentrations. Each panel includes the references of ACs (circle), while the other clones are indicated by squares. The dark gray, light gray and open symbols indicate the bottom 1%, mean and top 1% of the size distributions, respectively.
