# Supplementary figures and images for: Directed evolution of cell size in Escherichia coli
Source: BMC Evol Biol. 2014 Dec 17;14:257. doi: 10.1186/s12862-014-0257-1 (PMC4279887; doi:10.1186/s12862-014-0257-1)

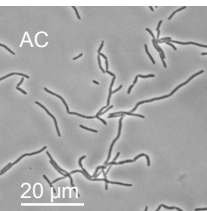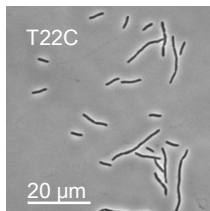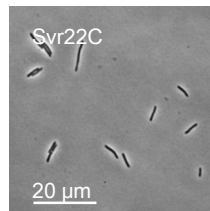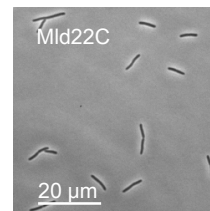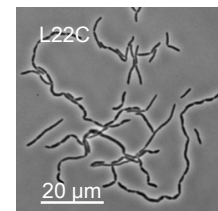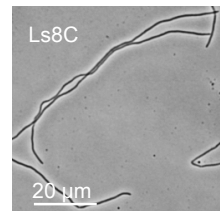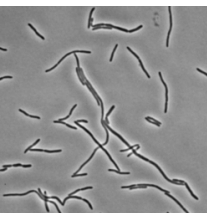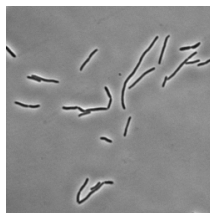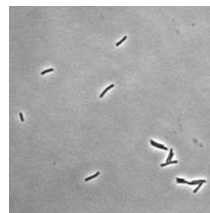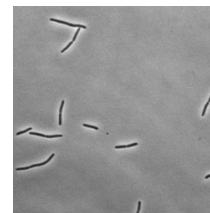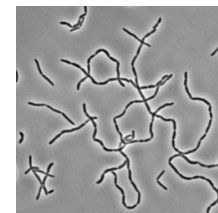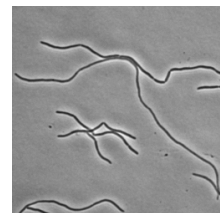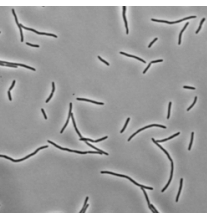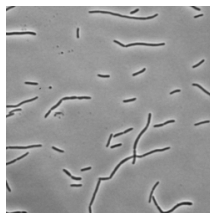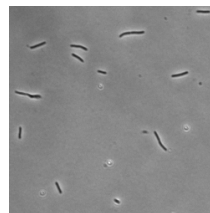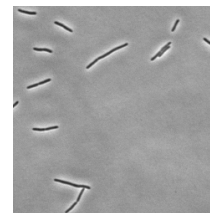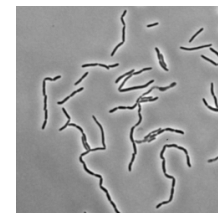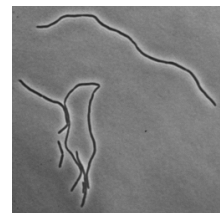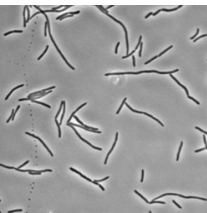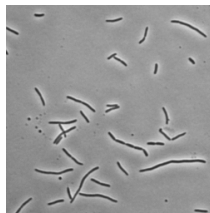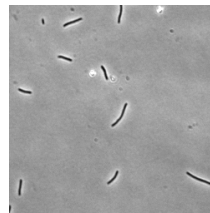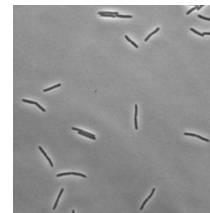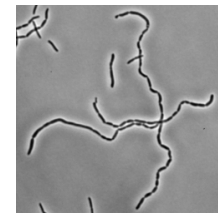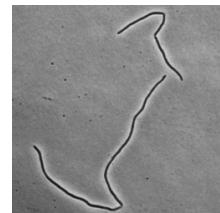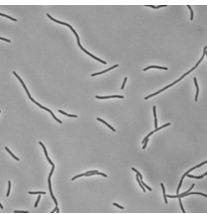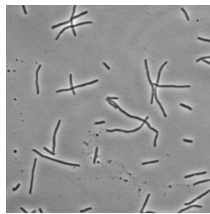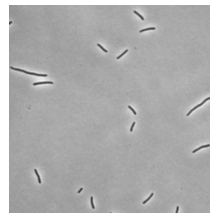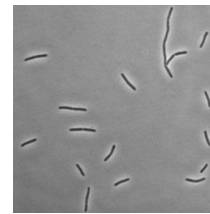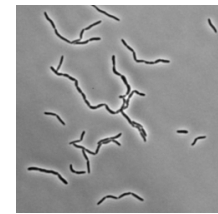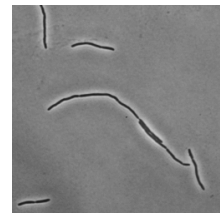

Supplement: Additional file 1: Figure S1. — Microscopic pictures of size-evolved cell populations. Each column corresponds to each cell population, as indicated in the top panels (AC, T22C, Svr22C, Mld22C L22C and Ls8C from left to right). These Cell IDs are explained in Table 1. Scale bars represent 20 μm. [file 12862_2014_257_MOESM1_ESM.pdf]

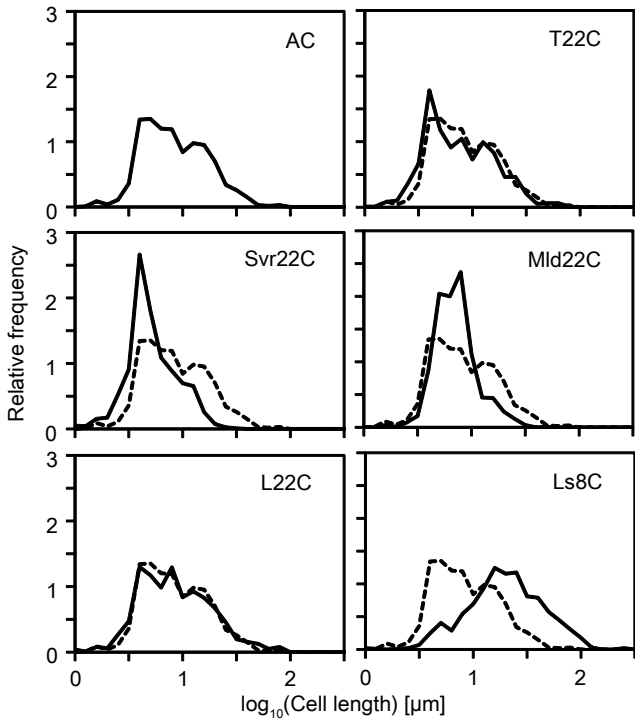

Supplement: Additional file 2: Figure S2. — Cell length distributions obtained from microscopic observation. Cell IDs are indicated in the insets. Dashed lines represent the distribution of AC (identical to top left panel) for reference. [file 12862_2014_257_MOESM2_ESM.pdf]

A

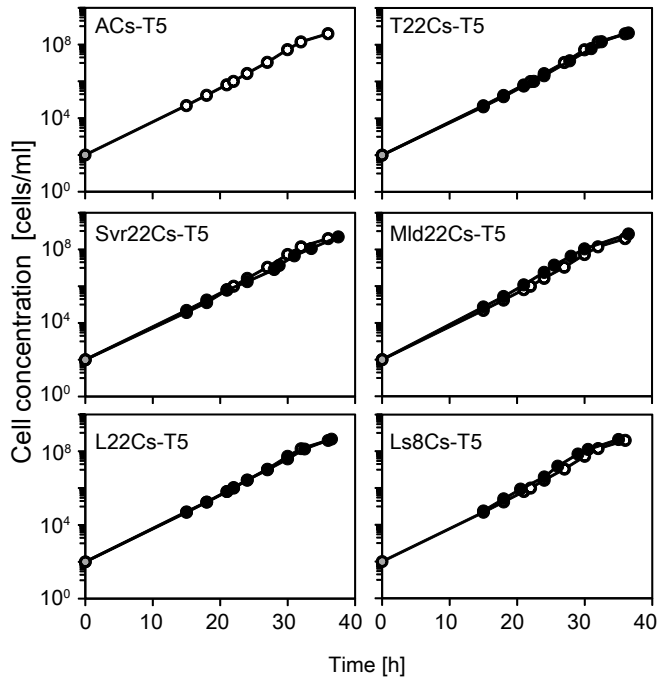

B

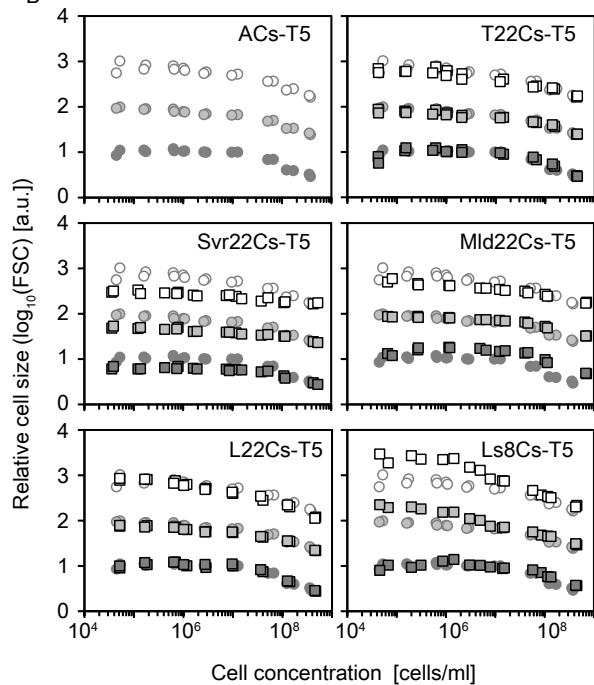

Supplement: Additional file 3: Figure S3. — The cell concentration dependency of cell size after daily serial transfers for 5 days without size selection. Twelve isolates from each evolved lineage (ACs, T22Cs, Svr22Cs, Mld22Cs, L22Cs and Ls8Cs) were propagated every day in the absence of size selection. In each propagation, 100 cells were transferred to 1 ml of fresh medium for 5 days (over 80 generations). The obtained populations are denoted as ACs-T5, T22Cs-T5, Svr22Cs-T5, Mld22Cs-T5, L22Cs-T5 and Ls8Cs-T5, as shown in the insets of the panels. (A) The mean growth curves of the transferred populations. Error bars indicate the standard deviation among the 12 isolates. The gray circles at time zero indicate the initial cell concentrations calculated from inoculation procedure. Each panel includes the growth curves of ACs (open circle), while the other clones are indicated by filled black circles. (B) The size references of the cell size distributions over different cell concentrations. Each panel includes the references of ACs (circle), while the other clones are indicated by squares. The dark gray, light gray and open symbols indicate the bottom 1%, mean and top 1% of the size distributions, respectively. [file 12862_2014_257_MOESM3_ESM.pdf]

Size selection via cell sorter

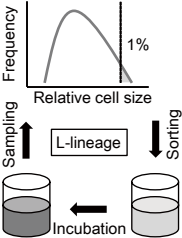

Growth selection in a culture

Supplement: Additional file 4: Figure S4. — Schematics of the experimental evolution toward a large cell size. Rounds for the directed size evolution consist of the particular size selections via cell sorter and the subsequent growth selections in a culture. The size selections were examined according to the fraction containing the largest 1% of cells (L-lineage). As a control, T-lineage consists of growth selection without size selection (as described in the main text). We determined the numbers of the sorted cells from the growth rate of the previous round, so they would reach approximately 107 cells/ml overnight. [file 12862_2014_257_MOESM4_ESM.pdf]

A

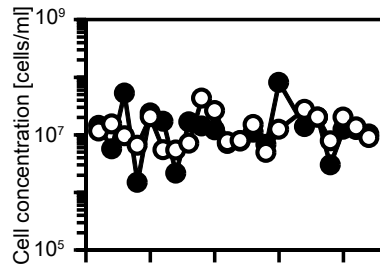

B

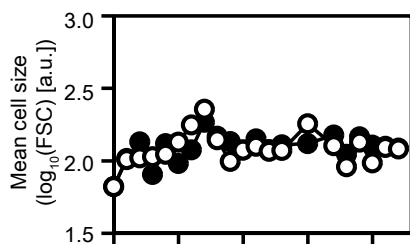

C

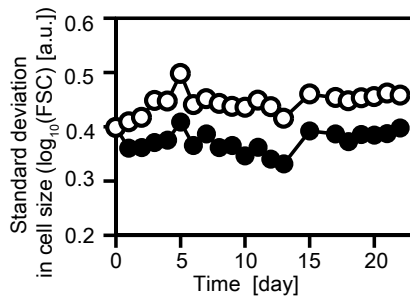

D

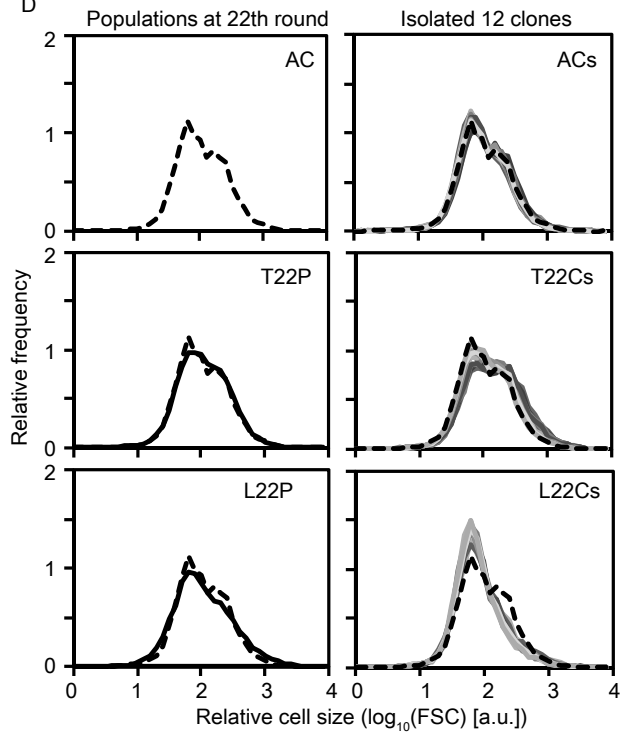

E

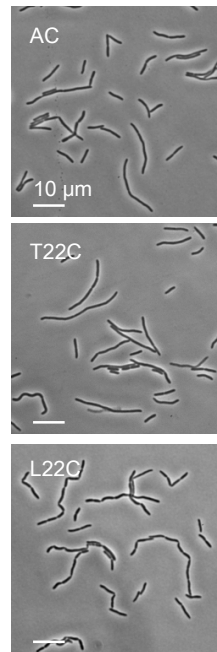

Supplement: Additional file 5: Figure S5. — Evolutionary process toward a large cell size. The trajectory of the cell concentration (A), the mean cell size (B) and the standard deviation (C) under the size selection process is shown. Open circles and closed circles represent the L- and T-lineages, respectively. (D) Cell size distributions of the population of the final round (left) and the isolated 12 clones (right), where the isolates are distinguished by gray-scaled lines. The top two panels represent ancestral clones. The other panels correspond to the T- and L-lineages at the bottom. The insets represent the Cell IDs in Table 1. All data were obtained at approximately 107 cells/ml. The dotted line indicates one of the AC isolated clones (top left). (E) Phase contrast images of the isolated clones. The insets also represent the Cell IDs in Table 1. The white bars indicate 10 μm. The other images are shown in Additional file 1: Figure S1. [file 12862_2014_257_MOESM5_ESM.pdf]

A

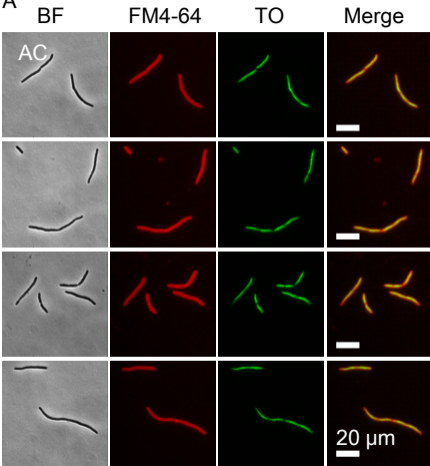

B

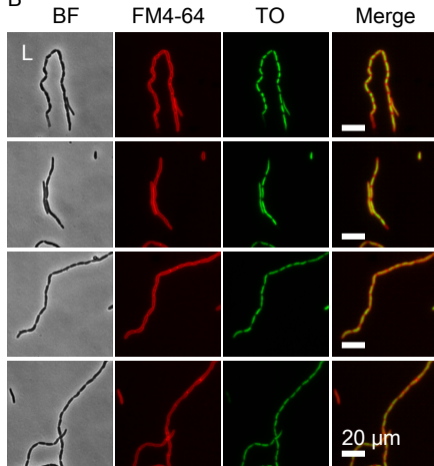

C

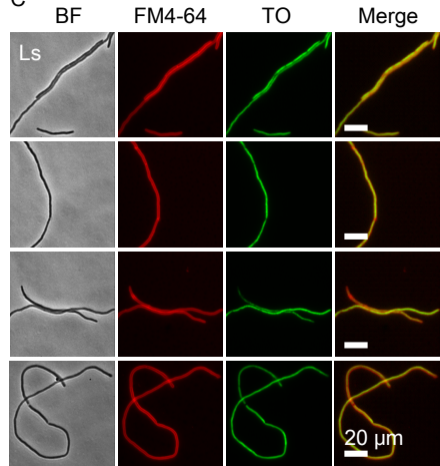

Supplement: Additional file 6: Figure S6. — Dividing septa of size-evolved cells. Phase contrast and fluorescence images of ACs (A), L22Cs (B) and Ls8Cs (C) are shown, where the Cell IDs are noted in Table 1. Cell membranes and DNA were stained with FM4-64 and Thiazole Orange (TO), respectively. For cell staining, cell cultures supplemented with 10 mM EDTA, 0.1% (v/v) Tween-20 and 420 pM of TO (BD Biosciences) were incubated for 15 min at room temperature. The cultures were further incubated for 1 min at room temperature after addition of 5 μg/ml of FM4-64 (Invitrogen). In each cell, bright field images (BF), red fluorescence images for FM4-64, green fluorescence images for TO and merged images (merged) were obtained (from left to right). The scale bars represent 20 μm. [file 12862_2014_257_MOESM6_ESM.pdf]

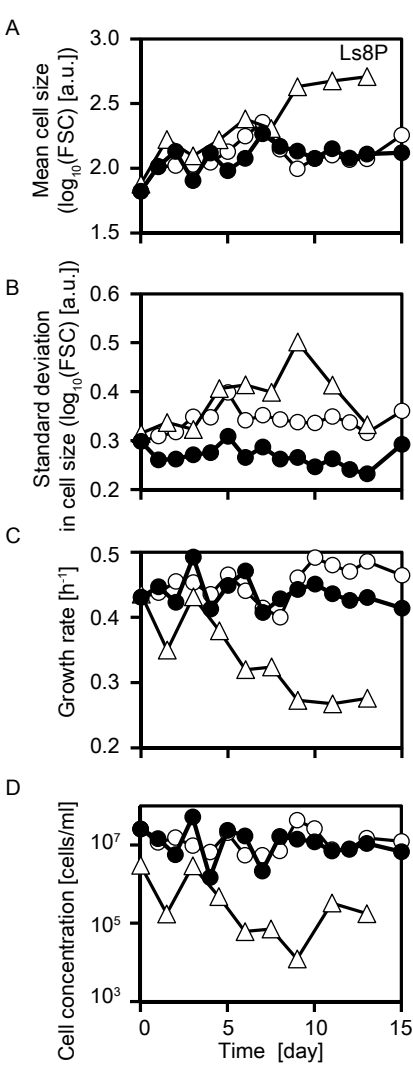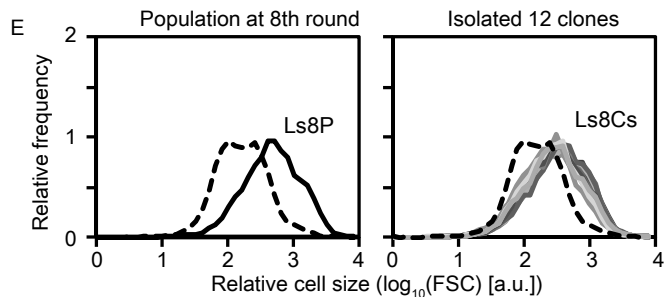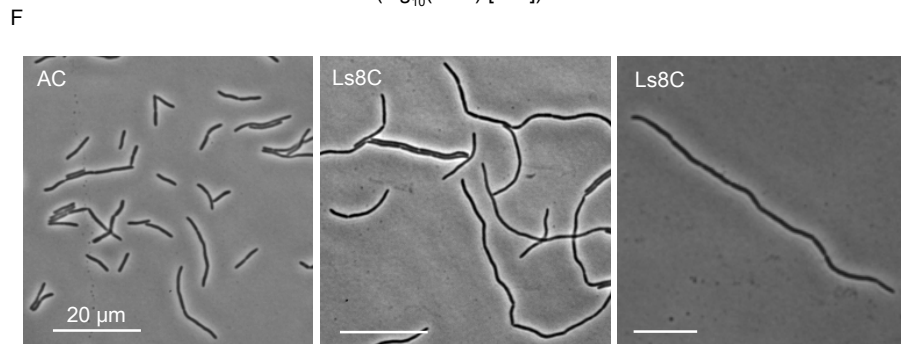

Supplement: Additional file 8: Figure S8. — Time-series behaviors in the selection cycles toward larger size through a single-cell bottleneck. The mean and standard deviation of the size distributions during the evolution process for the Ls-lineage (triangles) were measured (A and B), and the growth rates and the cell concentrations of the cultures were also calculated (C and D). The other circles are denoted in Additional file 7: Figure S7. The cell population at the final round (8th round) and its 12 isolates were analyzed to obtain the size distributions (E). The dotted line indicates one of the AC isolated clones in Additional file 5: Figure S5. The microscopic images of the evolved clone are shown with that of the ancestor (F). The scale bars represent 20 μm. The insets represent the Cell IDs in Table 1. [file 12862_2014_257_MOESM8_ESM.pdf]

A

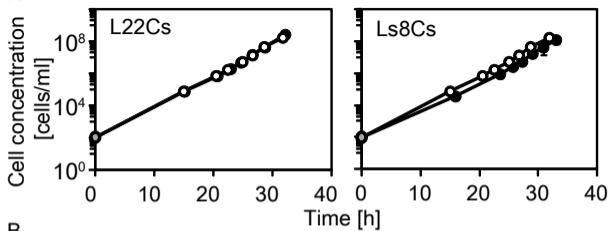

B

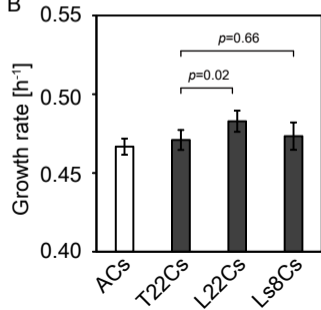

Supplement: Additional file 9: Figure S9. — Growth characteristics of the evolved populations toward larger size. (A) The averaged growth curves of the isolated 12 clones from L-lineage (L22Cs) and Ls-lineage (Ls8Cs). The Cell IDs are indicated in the inset. Open circles represent the ancestral clone (ACs). The gray circles at time zero indicate the estimated cell concentration from inoculation procedure. The error bars represent the standard deviation. (B) The growth rate of the isolated 12 clones for the exponential growth phase. The bars for ACs and T22Cs are shown for reference. The error bars represent 95% confidence intervals. P-values are for t-test. [file 12862_2014_257_MOESM9_ESM.pdf]

Relative cell size  
( $\log_{10}(\text{FSC})$  [a.u.])

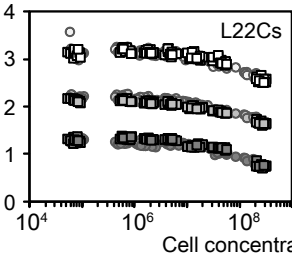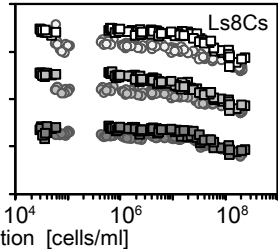

Supplement: Additional file 10: Figure S10 — The cell concentration dependency of cell size in the evolved populations toward larger size. The size references defined in Figure 4A of the cell size distributions over different cell concentrations. Each panel includes the references of ACs (circle), while the other clones are indicated by squares. The dark gray, light gray and open symbols indicate the bottom 1%, mean and top 1% of the size distributions, respectively. [file 12862_2014_257_MOESM10_ESM.pdf]
