# Supplementary material for: Directed evolution of cell size in Escherichia coli
Source: BMC Evol Biol. 2014 Dec 17;14:257. doi: 10.1186/s12862-014-0257-1 (PMC4279887; doi:10.1186/s12862-014-0257-1)
Supplement: Additional file 7: Figure S7. — Schematics of the experimental evolution to large cell size through a single-cell bottleneck. Seventy-two single cells in the largest 1% fraction in the population were sorted individually into the fresh medium (1 cell/ml) using 24-well plates. The cell cultures were incubated until the cell concentrations surpassed 104 cells/ml, which is the instrumental detection limit for cell number. The incubation time was 1.5 days until the 6th round and 2 days beyond that. The cell cultures for the subsequent cell sorting were selected among grown cultures based on which culture has the largest 1% size. Typically, approximately one-third of the cultures reached a sufficient cell concentration to be analyzed for the size selections in every round. The cells resulting from cycles toward the larger size through a single-cell bottleneck was called Ls-lineage. [file 12862_2014_257_MOESM7_ESM.pdf]

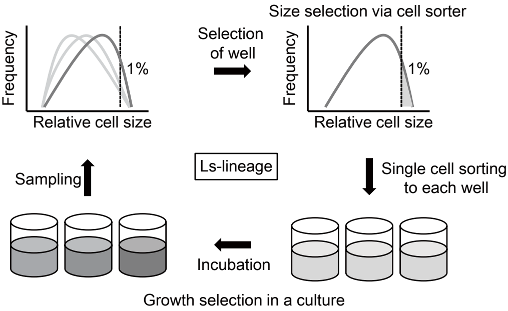
Size selection via cell sorter
Frequency
1%
Relative cell size
Selection
of well
Frequency
1%
Relative cell size
Ls-lineage
Sampling
Single cell sorting
to each well
Incubation
Growth selection in a culture
